# Tumor Lysis Syndrome in Patients with Hematological Malignancies

**DOI:** 10.1155/2017/9684909

**Published:** 2017-11-02

**Authors:** Yohannes Belay, Ketsela Yirdaw, Bamlaku Enawgaw

**Affiliations:** ^1^Ethiopian Public Health Institute (EPHI), Addis Ababa, Ethiopia; ^2^Department of Hematology & Immunohematology, School of Biomedical and Laboratory Sciences, College of Medicine and Health Sciences, University of Gondar, Gondar, Ethiopia; ^3^Department of Clinical Chemistry, School of Biomedical and Laboratory Sciences, College of Medicine and Health Sciences, University of Gondar, Gondar, Ethiopia

## Abstract

Tumor lysis syndrome is a metabolic complication that may follow the initiation of cancer therapy. It commonly occurs in hematological malignant patients particularly non-Hodgkin's lymphoma and acute leukemia due to chemotherapy or spontaneously. It is characterized by a biochemical abnormality such as hyperuricemia, hyperkalemia, hyperphosphatemia, and hypocalcemia and its clinical outcome is directly related to these biochemical abnormalities. Prevention and treatment of tumor lysis syndrome depend on immediate recognition of patients at risk. Therefore, identifying patients at risk and prophylactic measures are important to minimize the clinical consequences of tumor lysis syndrome. Patients with low risk should receive hydration and allopurinol. On the other hand patients with high risk should receive hydration and rasburicase in an inpatient setting. It is important to start therapy immediately, to correct all parameters before cancer treatment, to assess risk level of patients for TLS, and to select treatment options based on the risk level. In this review a comprehensive search of literatures was performed using MEDLINE/PubMed, Hinari, the Cochrane library, and Google Scholar to summarize diagnostic criteria, incidence, predicting factors, prevention, and treatment options for tumor lysis syndrome in patients with hematological malignancies.

## 1. Introduction

Tumor lysis syndrome (TLS) is a metabolic complication that may follow the initiation of cancer therapy. It is characterized by a metabolic abnormality including hyperuricemia, hyperkalemia, hyperphosphatemia, and hypocalcemia which occurs due to rapid lysis of tumor cells and leads to severe renal impairment, cardiac arrhythmia, or seizure and death [1, 2]. It is one of the oncologic emergency encounters in patients with hematological and other malignancies which causes death [3]. Cytolysis of cancerous cells can be caused by chemotherapy or it can occur spontaneously [4–6]. Spontaneous TLS is a rare occurrence but it may result in more severe clinical outcomes because of the lack of benefit of pretreatment [7].

Historically, TLS has been recognized in association with leukemia therapy and first reported by Bedrna and Polcák in chronic leukemia patients treated with irradiation in 1929 [8]. TLS is most frequently associated with hematological malignancies (HMs) such as lymphoma, leukemia, and multiple myeloma (MM) [4, 9]. Besides this, TLS also occurs in high proliferative and sensitive solid tumors [10].

This review summarizes the diagnostic criteria, pathophysiology and clinical presentation, incidence, risk factors, and prevention and treatment options of TLS in patients with HMs. For this purpose, a comprehensive search of literatures was performed using search terms like tumor lysis syndrome, incidence, hematological malignancy, risk factor, electrolyte abnormality, uric acid, allopurinol, rasburicase, febuxostat, and combination of these terms by using MEDLINE/PubMed, Hinari, the Cochrane library, and Google Scholar. The relevant publications based on searches were examined for this narrative review preparation. In addition to full articles, abstracts available related to TLS were also included.

## 2. Definition and Classification of Tumor Lysis Syndrome

Although there is a general consensus for a broad definition of TLS as a set of metabolic complications that can occur in rapidly proliferating neoplasm after anticancer therapy initiation, there is no universal and standard diagnostic definition and classification system for TLS. In 1993, Hande and Garrow published the first formal definition [1]. However, it has some limitations and was modified by Cairo and Bishop in 2004 [2] to formulate a uniformly used classification system for TLS. Based on the Cairo and Bishop definition, TLS can be classified as laboratory or clinical TLS. Laboratory defined TLS is a type of TLS which is characterized by biochemical changes without clinical manifestation. Patients can have severe metabolic derangements without symptoms. These require treatment. Clinical TLS is defined as biochemical changes which are accompanied by clinical features and need urgent management [2].

Cairo and Bishop defined laboratory TLS (LTLS) as an abnormality of two or more of the following, occurring up to three days before or seven days after chemotherapy: uric acid (UA) ≥ 476 mmol/L, potassium ≥ 6.0 mmol/L, phosphorus ≥ 2.1 mmol/L in children and ≥1.45 mmol/L in adults, and calcium ≤ 1.75 mmol/L or 25% increase from baseline for UA, potassium, and phosphorus and 25% decrease from baseline for calcium. On the other hand, clinical TLS (CTLS) is defined as a LTLS with one or more of the following abnormalities: creatinine more than 1.5 times upper limit normal (ULN), cardiac arrhythmia/sudden death, and seizure [2].

The above definition of the Cairo and Bishop for TLS is important to identify patients with laboratory evidence of TLS but who do not require a specific therapeutic intervention from those patients who experienced life-threatening clinical abnormalities that required a specific intervention (e.g., dialysis). However, the Cairo and Bishop classification system has some shortcomings. Patients developing TLS may not always have two or more abnormalities present at once, but one metabolic derangement may precede another abnormality. A 25% increase/decrease from baseline may not always be significant if it does not result in a value outside the normal range. Since hypocalcemia may not be considered as a direct consequence of TLS and is associated with high phosphate levels, it is difficult to include as diagnostic criteria.

In 2011, Howard et al. modify the Cairo & Bishop TLS classification by omitting the need for a 25% change laboratory values from the baseline. According to Howard and his colleagues, the 25% change from the baseline is not clinically important, and they defined LTLS as presentation of two or more metabolic abnormalities during the same 24-hour period within 3 days before to 7 days after initiation of therapy. On the other hand, LTLS plus an increased creatinine level, seizures, cardiac dysrhythmia, or death constitutes CTLS [11].

A uniform diagnosis criterion is important to determine the exact incidence and risk classification of TLS. It becomes more relevant for comparisons of new therapies for the prevention and prophylaxis of TLS in the future. Even if there is no uniformly accepted diagnostic and classification system for TLS, the Cairo and Bishop diagnostic definition is often used. The lack of universal definitions for diagnosis has made the analysis of different studies examining TLS complicated because of heterogeneity of diagnostic criteria, the variability of patient study duration, variations in prophylactic treatments, and broad variations in the type of therapy. It makes comparisons between studies difficult [4, 12–15].

## 3. Pathophysiology and Clinical Presentation of Tumor Lysis Syndrome

The main pathophysiology of TLS lies in the fact that malignant cells are rich in purines, potassium, and phosphorus. When these cells die spontaneously or secondary to therapy, intracellular substances are released to extracellular fluid and mediate the pathophysiology of TLS and its clinical complications. The increase in the concentration of potassium, UA, and phosphate affects the normal homeostatic mechanisms of the body and can result in numerous metabolic derangements, impaired organ function, and associated morbidity. The clinical presentation/outcome of TLS is directly linked to the biochemical abnormalities which include hypocalcemia, hyperkalemia, hyperphosphatemia, and hyperuricemia. Several symptoms and abnormalities may appear due to TLS including renal, cardiac, neurological, and muscular manifestations. Although the signs and symptoms of TLS may occur as early as a few hours after the start of chemotherapy, they are more common 24 to 48 hours following initiation of treatment [2, 16]. The pathophysiology and clinical presentation of TLS depend on the combination of individual biochemical abnormalities in the patient.

Cell lysis results in the release of enormous amounts of potassium, which is concentrated intracellularly, into extracellular fluid and results in hyperkalemia, which is one of the key laboratory manifestations of TLS. In addition, an early peak in serum concentration may appear because of stress due to radiotherapy or chemotherapy that may reduce adenosine triphosphate levels and result in the release of potassium before complete lysis of tumor cells [17, 18]. Hyperkalemia is most severe component of TLS and may appear from 6 to 72 hours after chemotherapy initiation [17, 19]. Hyperkalemia can affect the skeletal muscle and cardiac myocardium. Patients with hyperkalemia present with fatigue, electrocardiogram abnormalities, and serious cardiac arrhythmias including cardiac arrest. Severe hyperkalemia can adversely affect skeletal and cardiac muscle function. ECG changes include widening of the QRS complex and peaked T waves. If severe hyperkalemia is not treated properly and timely, it results in sudden death as a result of cardiac dysrhythmia [20, 21]. Hyperkalemia must be corrected rapidly before potentially fatal ventricular arrhythmias occurred [22, 23].

Uric acid is a terminal product of the purine nucleotides adenine and guanine in humans, which constitute the backbone of nucleic acids. Purines are metabolized to hypoxanthine and xanthine via the action of enzymatic activity of xanthine oxidase to UA [24, 25]. The rate of UA clearance is highly dependent on the flow rate of the glomerular filtrate through the renal tubule. Because of a high cellular turnover in malignancy both spontaneously or due to therapy, large amounts of purines are released and lead to a rapid increase of UA (hyperuricemia). Hyperuricemia develops from 48 to 72 hours after treatment initiation and results in UA nephropathy form pathological urate crystal deposition in the renal tubules resulting in acute kidney injury (AKI) [16, 19, 26]. It is the main cause of renal failure [27]. AKI is the most common manifestation of TLS [28]. Its incidence in patients with HMs varies from 30% to 69% depending on the relative proportions of patients with leukemia, lymphoma, and MM [29–32].

The third metabolic abnormalities related to TLS is hyperphosphatemia. Hyperphosphatemia may develop from 24 to 48 hours after treatment. The release of intracellular phosphate overwhelms the normal renal threshold for phosphate excretion and results in hyperphosphatemia. Malignant hematologic cells may contain up to four times more intracellular phosphate compared with normal mature lymphoid cells [19]. Patients with spontaneous TLS may have lower rates of hyperphosphatemia due to phosphate uptake into rapidly dividing tumor cells compared to therapy-related TLS [7, 25]. Acute destruction of tumor cells during chemotherapy prevents the rapid reuse of phosphate for newly synthesized tumor cells. When in excess, phosphorus tends to bind to calcium and form calcium phosphate [2, 7]. The formation of calcium phosphate precipitates in the renal tubules which leads to acute renal failure which is the most significant complication resulting from hyperphosphatemia. Hyperphosphatemia may also result in muscle cramps, tetany, cardiac arrhythmia, and seizures [2, 16, 33, 34].

The other complication related to TLS is hypocalcemia. It is frequently found in association with hyperphosphatemia since phosphorus and calcium homeostasis are closely and reciprocally linked [2, 16]. Hypocalcemia may present with phosphate calcium crystal deposition and is rarely symptomatic. However, it may present with symptoms as nausea, vomiting, muscular hyperactivation such as spasms and tetany, seizures, prolongation of QT interval on the ECG, cardiac dysrhythmias, and alterations of mental status [34, 35].

In general, hyperkalemia is often the earliest laboratory manifestation. Hyperkalemia and hyperphosphatemia result directly from rapid malignant cell lysis. Hypocalcemia is a consequence of acute hyperphosphatemia with subsequent precipitation of calcium phosphate in soft tissues and UA is the end product of purines metabolism in humans. Although AKI remains the commonest, neurological or cardiac manifestations of TLS remain rare.

## 4. Incidence of Tumor Lysis Syndrome in Hematological Malignancies 

The incidence of TLS varies widely in HMs depending on the underlying malignancy and the definition of TLS. It ranges from case reports in certain chronic malignancies to 45% incidence reported in children with acute lymphoblastic leukemia (ALL) which depends on patient's risk factors and diagnostic criteria [6, 14, 36]. TLS usually develops after initiation of chemotherapy but there are also TLS occurring spontaneously, that is, not requiring initiation of therapy. Spontaneous TLS is typically observed in high-grade HMs such as B-cell non-Hodgkin lymphoma (NHL) [7, 37, 38].

TLS occurs most frequently in high-grade NHL and acute leukemia and less commonly in chronic leukemia and MM. This is supported by the following findings conducted in different facilities in children and adult age groups. Wasim et al. studied 50 patients diagnosed with HMs to determine the frequency of TLS and reported the incidence of 14%, 4%, and 2% for acute leukemia, NHL, and chronic leukemia, respectively [39]. In a multicenter cohort study of 153 high-risk patients with acute leukemia, aggressive NHL, and Burkitt leukemia/lymphoma, the overall incidence of TLS was found to be 30.7% [32]. Sevinir et al. reviewed medical records of 327 children with NHL and ALL retrospectively and reported overall 5.8% incidence of TLS and an incidence of 15.9 % and 0.47% in NHL and ALL, respectively [40]. In another review of 398 children diagnosed with ALL conducted by Al Bagshi et al., the TLS incidence of 19% has been reported based on Hande and Garrow definitions [12]. Bahoush et al. conducted a study on 160 children with ALL to identify children with low risk for TLS and reported a 26% incidence of TLS [13]. Ahsan Ejaz et al. also reported 32% incidence of TLS in acute myeloid leukemia (AML) patients using retrospective analysis of 183 patients [41].

A case report of TLS has been reported in chronic leukemia and MM. Occasionally, in these malignancies, highest incidence of TLS has been reported depending on the chemotherapy undertaken. Blum et al. studied 116 patients with chronic lymphocytic leukemia (CLL) treated with the cyclin-dependent kinase inhibitor, flavopiridol, and reported 46% incidence of TLS [15]. In a retrospective study conducted by Cairo et al. on 951 patients diagnosed with cancer highest rate of TLS (42%) has been reported in MM among HMs [9].

## 5. Risk Factors and Predictors of Tumor Lysis Syndrome 

TLS occurs more frequently in HMs than in solid tumors. The highest risk of developing TLS is observed in patients with lymphoproliferative disorders with high proliferative rate and high tumor sensitivity to chemotherapy, like B-cell ALL and Burkitt's lymphoma [42, 43]. Tumor burden, reflected by serum lactate dehydrogenase (LDH) level, initial white blood cell count (WBC), tumor size, and extensive bone marrow involvement are the main predictor for development of TLS in these patients [2]. When assessing the risk of TLS in a particular patient, it is important to consider both the patient and tumor related predictors of risk. The risk of developing TLS is influenced by a number of characteristics including tumor, patient, and therapy specific factors [11, 44]. Specific risk factors are discussed below.

### 5.1. Patient Related Factors

Patient related factors such as dehydration status, advanced age, presence of splenomegaly, presence of mediastinal mass, central nervous system (CNS) and renal involvement, increased baseline creatinine level, increased UA level, elevated LDH level, high WBC count, and impaired kidney function can affect the development of TLS [12, 14, 32, 45]. Older age is related to decreased renal function and leads to a reduction in the glomerular filtration rate. This decreased urine output leads to increasing concentration of metabolites in blood [46]. A study conducted to identify children with ALL at low risk for TLS reported that CNS involvement, renal involvement, presence of mediastinal mass, and baseline WBC count ≥20 × 10^9^/L were independent predicting factors with odds ratio of 11.6, 8.1, 4.3, and 3.1, respectively [13]. Another study conducted on AML patients using multivariate analysis showed that WBC count > 25 × 10^9^/L, LDH level above ULN, UA > 7.5 mg/dl, and creatinine > 1.4 mg/dl were identified as independent risk factors for TLS development [28]. Truong et al. reviewed 398 patients aged ≤ 18 years diagnosed with ALL and reported that splenomegaly, mediastinal mass, and initial WBC > 20 × 10^9^/L were found to be independent predictors of TLS [47]. In a pilot trial that investigated the safety of rituximab in children with advanced mature B-NHL, patients with high LDH had higher incidence of TLS and there was a strong association between TLS development and high initial LDH, with 13% of those with LDH < 2 × ULN versus 45% of those with high LDH (≥2 × ULN) developing TLS [48].

### 5.2. Tumor Related Factors

Malignancies with high cancer mass, bulky disease, large tumor burden, sensitivity of the malignancy to chemotherapy, cancer stage, and rapid proliferation rate of malignant cells are tumor specific factors related to TLS [11, 41, 43, 44]. Increased tumor burden is most specific cancer risk factor which is demonstrated by large size, elevated LDH, and increased WBC count. The quantity of cellular contents released after the administration of effective chemotherapy is increased when there is greater cancer mass [43]. HMs with a high potential for cell lysis include high-grade lymphomas and acute leukemias [32, 39]. A multicenter study of children and adolescents up to 18 years of age with NHL indicated that patients with Burkitt's lymphoma or B-ALL had the highest incidence of TLS (8.4%) compared with other NHL which was below 2%, suggesting that patients with Burkitt's lymphoma or B-ALL were at the highest risk of developing TLS [42].

### 5.3. Therapy-Related Factors

Intensive polychemotherapy, corticosteroids, intrathecal chemotherapy [5], radiotherapy, and interferon [44] are cytotoxic therapies more frequently associated with TLS. The use of certain cytotoxic agents such as combination therapy with bortezomib, cyclophosphamide, and dexamethasone in MM patients [49, 50] and ibrutinib, fludarabine, and rituximab in CLL [15, 36] may result in TLS. The introduction of more aggressive chemotherapy in the management of HMs may contribute to an increase in incidence of TLS. One study conducted to determine frequency of TLS in aggressive and slow introduction chemotherapy in children with ALL indicated that greater number of patients developedTLS in aggressive compared to slow induction chemotherapy (7/10 in aggressive and 3/10 in slow chemotherapy) [51].

### 5.4. Risk Classification of TLS

Cairo et al. proposed TLS risk classification system that combines multiple factors into assessment of the patient's risk of developing TLS. Based on this risk classification system, a patient has no TLS at the time of presentation categorized into three groups based on patient related factors (preexisting renal) and disease related factors (tumor type, tumor burden which is represented by tumor stage, WBC counts, and LDH levels) (Table 1) [43].

Patients with lymphomas or leukemias considered to be low risk disease were classified as being at an intermediate risk of developing TLS if there was renal dysfunction and/or renal involvement. Similarly, patients with leukemias and lymphomas considered to be low risk disease were classified as being at a high-risk of developing TLS if there was renal dysfunction and/or renal involvement. Patients with low risk disease and normal renal function would also be high-risk for TLS if UA, phosphate, or potassium levels were elevated [43].

Patients at low and intermediate risk can be treated with hydration and allopurinol while high-risk patients are treated with rasburicase and increased hydration [43, 52].

## 6. Prevention and Treatment

TLS prophylaxis is recommended to all patients with HMs undergoing chemotherapy. Recognizing risk factors is an important step to prevent TLS. It is important to minimize or eliminate factors that may result in a greater risk of TLS. Prevention is the best treatment for TLS. Treatment and prevention of TLS consist of using medications that decrease UA (hypouricemic agents), electrolyte management, and adequate hydration before and following chemotherapy [11, 44, 53]. Patients with low risk should receive hydration and allopurinol. Patients at high risk should receive hydration and rasburicase in an inpatient setting. In order to avoid xanthine accumulation and lack of substrate for rasburicase, concomitant allopurinol should not be administered [44].

### 6.1. Hydration

Adequate hydration is the first measure undertaken to increase intravascular volume and preventing TLS. This decreases extracellular concentrations of UA, phosphorus, and potassium and enhances renal blood flow to maintain sufficient glomerular filtration rate and urine output. When possible, intravenous hydration should be started at least 24 to 48 hours prior to chemotherapy initiation and continued during chemotherapy, depending on tumor type or the patient's clinical condition. In patients with underlying acute kidney injury or cardiac dysfunction, IV hydration can lead to potentially dangerous fluid overload. In this occasion, close monitoring and follow-up are mandatory. If adequate urine output cannot be achieved with IV hydration alone, diuretics may be important to achieve the desired outcome [2, 16, 54]. Since these diuretics may contribute uric acid or calcium phosphate precipitation in renal tubules, the patient must be well hydrated.

### 6.2. Hypouricemic Agents

#### 6.2.1. Allopurinol

Allopurinol is begun 2-3 days prior to chemotherapy and continued for 10–14 days as a first-line treatment of hyperuricemia. It is a competitive inhibitor of xanthine oxidase, which is an enzyme used to break down hypoxanthine to xanthine and xanthine to UA. Although allopurinol is effective at inhibiting new UA formation, it is not effective in reducing existing UA level. However, inhibition of xanthine oxidase may result in a buildup of xanthine and hypoxanthine concentration, which is less soluble than UA, and form crystals that deposit in the kidney leading to xanthine nephropathy [55, 56]. The effect of allopurinol is relatively slow, taking several days to produce a reduction in UA level compared to rasburicase. A multicenter study conducted on adults at risk for TLS demonstrated that allopurinol had significantly lower response rate for UA than rasburicase (66% versus 87%). The mean UA reduction within four hours was also lower in allopurinol (14%) than rasburicase (88%) [57].

#### 6.2.2. Rasburicase

Rasburicase is begun when allopurinol is ineffective for hyperuricemia treatment. It is a pure recombinant form of urate oxidase, an enzyme responsible for the breakdown of UA into allantoin, which is easily soluble and readily removed [58]. Rasburicase is safe and effective in decreasing serum UA levels in lymphoma and leukemia patients undertaking chemotherapy [48, 57–61]. Rasburicase can be used as hypouricemic therapy with hydration in high-risk patient and patients with laboratory and clinical TLS [43, 44]. The primary advantage of urate oxidase is its rapid start of the action. It rapidly lowers UA levels, usually within four hours of administration. It provides better control of UA compared to allopurinol. A randomized study conducted to compare rasburicase and allopurinol in children with lymphoma and leukemia indicated that patients randomized to rasburicase had better reduction of initial plasma UA levels compared to allopurinol in four hours after the first dose (86% versus 12% reduction). In this study the rasburicase group (AUC_0–96_ of 128 ± 70 mg/dL hour) experienced 2.6-fold less exposure to UA compared to allopurinol group (329 ± 129 mg/dL hour) [58]. Another multicenter study conducted to evaluate safety and efficacy of rasburicase showed that 99% of hyperuricemic patients responded to rasburicase treatment. Similarly all patients (nonhyperuricemic) who received rasburicase prophylactically maintained low uric acid levels despite ongoing chemotherapy [62]. One of the disadvantages of rasburicase is its high cost [63]. Secondly, it should not be used in patients with glucose-6-phosphate dehydrogenase (G6PD) deficiency due to the risk of hemolysis following its administration. Rasburicase breaks down UA and results in the production of hydrogen peroxide. In patients with G6PD deficiency, it may result in an increased risk of hemolytic anemia and methemoglobinemia [64–66].

#### 6.2.3. Febuxostat

Febuxostat is a novel new nonpurine selective inhibitor of xanthine oxidase, which is a promising alternative to allopurinol in patients who are unable to tolerate allopurinol, have inadequate response to allopurinol, or have renal dysfunction. It decreases and maintains serum UA levels more effectively than allopurinol in patients with hyperuricemia. It is more effective than allopurinol in patients with impaired renal function. Febuxostat was safe and effective in preventing or reversing hyperuricemia in patients with HMs who were undergoing chemotherapy and at an intermediate risk to develop TLS [67–69]. Schumacher et al. conducted randomized and double blinded trial to study the effect of febuxostat and allopurinol in subjects with hyperuricemia and gout and reported that significantly higher percentages of subjects treated with febuxostat 240 mg (69%) attained serum urate levels < 6.0 mg/dl compared with allopurinol (22%). About 60% of subjects with impaired renal function treated with febuxostat 240 mg achieved < 6.0 mg/dl compared with those treated with 100 mg of allopurinol (0%) [70].

### 6.3. Urine Alkalinization

Alkalinization is commonly used as part of the preventative measure of TLS, since this increases the urine pH which makes UA more soluble and less likely to precipitate in the renal tubules. However, alkalinizing the urine may facilitate calcium phosphate deposition in malignant patients with severe hyperphosphatemia [71]. Urinary alkalization can cause a xanthine nephropathy by decreasing the solubility of xanthine, a precursor of UA. Alkalinization of the urine assists in decreasing the incidence of UA nephropathy and subsequent renal failure by reducing UA crystallization. In alkaline environments, UA remains ionized. Thus, it is more water soluble and more readily excreted by the kidneys [2, 16]. However, alkalinization of the urine, once a common treatment for TLS, is no longer routinely recommended because it may be associated with metabolic acidosis and calcium phosphate precipitation [25, 72].

### 6.4. Electrolyte Management

Another measure is the discontinuation of agents that may worsen the patient's condition with experience of TLS. Electrolyte supplementation, particularly potassium and phosphorus products, should be stopped and removed from IV fluids to avoid the risk of worsening electrolyte abnormalities. Hyperphosphatemia is managed with phosphate binders such as aluminum hydroxide which will decrease the gut absorption of phosphate. Usually, treatments of hyperphosphatemia will self-correct any related hypocalcemia. Calcium itself should not be administered as it may precipitate metastatic calcifications [23, 73, 74].

Mild hyperkalemia can be managed with sodium polystyrene sulfonate. Treatments for severe hyperkalemia with or without ECG changes include hypertonic glucose and insulin, loop diuretics, and bicarbonate. Hypertonic glucose and insulin will shift potassium from the extracellular to the intracellular space. Sodium bicarbonate shifts potassium intracellularly and a slight alkalinization favors distal tubule secretion as well. Loop diuretics promote potassium excretion [22, 73].

### 6.5. Dialysis

A patient who does not respond to the above measures may need renal replacement therapy, such as hemodialysis, to manage electrolyte abnormalities and hyperuricemia and treat renal failure associated with TLS. Hemodialysis should be considered for every patient with excessively elevated UA, phosphate, and/or potassium not responsive to pharmacologic intervention and in those patients in whom acute renal failure develops despite prevention to control volume overload, electrolyte abnormalities, and uremia [72]. Dialysis may be initiated prophylactically before the development of overt uremic symptoms in response to severe, progressive hyperphosphatemia or severe symptomatic hypocalcemia. Frequent dialysis is recommended considering the continuous release into the bloodstream of purine products, potassium, and other metabolites and electrolytes resulting from lysed tumor cells. The timing of dialysis and the dialysis dose should be linked to the purine generation rate [44, 75].

## 7. Conclusion and Recommendation

Tumor lysis syndrome is a common and life-threatening event in patients with lymphoma and leukemia undertaking chemotherapy. The incidence is increasing because of more effective cancer treatments and needs due attention in these malignancies. There must be standard and universally accepted diagnostic criteria for TLS to start therapy immediately, to correct all parameters before cancer treatment, to assess risk level of patients for TLS, and to select treatment options based on the risk level. If the patient is at low and intermediate risk, IV fluid with allopurinol is given with close monitoring while high-risk patients are treated with rasburicase and increased hydration. Dialysis is the last option if the other treatment options failed. Since TLS is potentially fatal, close monitoring of patients at risk before, during, and after their course of chemotherapy is critical. Identifying patients at risk and prophylactic measures are important to minimize the clinical consequences of this syndrome.

## Figures and Tables

**Table 1:** Patients risk classification in HMs.

Risk group	Type of HMs
High risk	(i) Advanced stage Burkitt's lymphoma/leukemia (B-ALL)
	(ii) Lymphoblastic lymphoma with LDH ≥ 2 × ULN
	(iii) Early stage Burkitt's lymphoma/leukemia with LDH ≥ 2 × ULN
	(iv) ALL with WBC count ≥ 100 × 10^9^/L or less if the baseline elevation of LDH is twice ULN (v) AML with WBC count ≥100 × 10^9^/L
	(vi) Diffuse large B-cell lymphoma (DLBCL) with an elevated baseline LDH of twice ULN, and bulky disease

Intermediate risk	(i) AML with a WBC between 25 × 10^9^/l and 100 × 10^9^/l or <25 × 10^9^/L if the baseline elevation of LDH is twice ULN
	(ii) ALL with WBC < 100 × 10^9^/L and an LDH of less than twice ULN
	(iii) Early stage Burkitt lymphoma/leukemia with an LDH of less than twice ULN
	(iv) DLBCL with a baseline increase in LDH of twice ULN but nonbulky disease
	(v) CLL with WBC ≥ 50 × 10^9^/L

Low risk	(i) Indolent lymphomas
	(ii) CLL
	(iii) Chronic myelogenous leukemia (CML)
	(iv) AML with WBC count < 25 × 10^9^/L and an LDH elevated to less than twice ULN
	(v) MM

*Source*. Customized from recommendations for the evaluation of risk and prophylaxis of tumor lysis syndrome (TLS) in adults and children with malignant diseases: an expert TLS panel consensus. BJH, Cairo MS et al. 149, 578–586, copyright 2010 Blackwell Publishing Ltd.

## Abbreviations

AKI: Acute kidney injury
ALL: Acute lymphoblastic leukemia
AML: Acute myeloid leukemia
CLL: Chronic lymphocytic leukemia
CML: Chronic myelogenous leukemia
CNS: Central nerves system
CTLS: Clinical tumor lysis syndrome
DLBCL: Diffuse large B-cell lymphoma
ECG: Electrocardiogram
G6PD: Glucose-6-phosphate dehydrogenase
HMs: Hematological malignancies
IV: Intravenous
LDH: Lactate dehydrogenase
LTLS: Laboratory tumor lysis syndrome
MM: Multiple myeloma
NHL: Non-Hodgkin's lymphoma
TLS: Tumor lysis syndrome
UA: Uric acid
ULN: Upper limit normal
WBC: White blood cell
AUC: Area under the curve.
